# DNA Methylation Change Profiling of Colorectal Disease: Screening towards Clinical Use

**DOI:** 10.3390/life11050412

**Published:** 2021-04-30

**Authors:** Joo Mi Yi

**Affiliations:** 1 Department of Microbiology and Immunology, College of Medicine, Inje University, Busan 47392, Korea; jmyi76@inje.ac.kr; 2 Innovative Therapeutics Research Institute, College of Medicine, Inje University, Busan 47392, Korea

**Keywords:** epigenetic regulation, DNA methylation, colorectal cancer, inflammatory bowel diseases (IBDs), biomarkers

## Abstract

Colon cancer remains one of the leading causes of cancer-related deaths worldwide. Transformation of colon epithelial cells into invasive adenocarcinomas has been well known to be due to the accumulation of multiple genetic and epigenetic changes. In the past decade, the etiology of inflammatory bowel disease (IBD) which is characterized by chronic inflammation of the intestinal mucosa, was only partially explained by genetic studies providing susceptibility loci, but recently epigenetic studies have provided critical evidences affecting IBD pathogenesis. Over the past decade, A deep understanding of epigenetics along with technological advances have led to identifying numerous genes that are regulated by promoter DNA hypermethylation in colorectal diseases. Recent advances in our understanding of the role of DNA methylation in colorectal diseases could improve a multitude of powerful DNA methylation-based biomarkers, particularly for use as diagnosis, prognosis, and prediction for therapeutic approaches. This review focuses on the emerging potential for translational research of epigenetic alterations into clinical utility as molecular biomarkers. Moreover, this review discusses recent progress regarding the identification of unknown hypermethylated genes in colon cancers and IBD, as well as their possible role in clinical practice, which will have important clinical significance, particularly in the era of the personalized medicine.

## 1. Introduction

Epigenetics have been defined as the mechanisms that initiate and maintain heritable patters of gene function and regulation in a friable manner without affecting the sequence of the genome. There are three main mechanistic layers in the field of epigenetic alterations, which include DNA methylation, histone modification, and microRNAs [1]. Epigenetic regulation has recently been highlighted as a prospective mechanism of cancer therapy. Therefore, an understanding of epigenetic mechanisms in cancer is required to improve epigenetic therapies based on biological significance such as gene interactions, regulation of pathways, and the function of epigenetic changes. The biological roles of epigenetic components in cancer development, called the “cancer epigenome,” have led to new opportunities for understanding the process of cancer therapy, including the detection, treatment, and prevention of cancer. However, this concept of the epigenome contributing to the understanding of cancer development has recently expanded to other human diseases, such as inflammatory bowel disease (IBD). Therefore, genome-wide methylation profiling studies provide an entirely new approach to understanding the importance of DNA methylation at the global transcriptional level during cancer development [2]. This review outlines recent genome-wide epigenetic discoveries in colorectal cancer and IBD with a focus on the roles of how the epigenome may contribute to detecting or preventing cancer or other human diseases for further translational applications.

## 2. Epigenetic Regulation in Human Cancers 

### 2.1. Histone Modification in Cancer 

**Figure 1:**
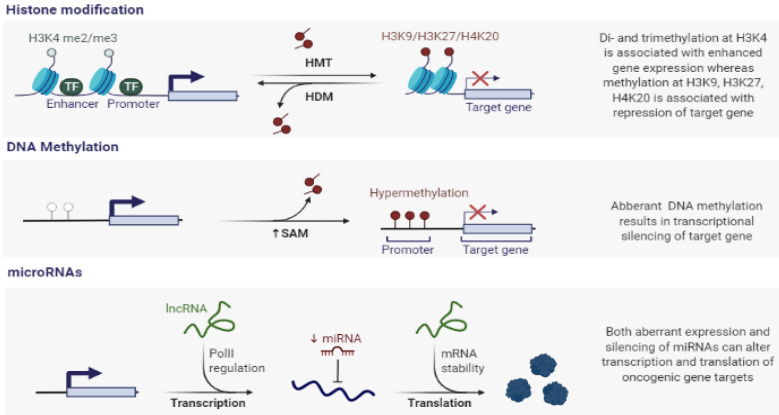
Schematics of the main epigenetic mechanisms associated with gene transcriptional silencing. Histone modifications, DNA methylation, and non-coding RNA mediated gene silencing constitute three distinct mechanisms of epigenetic regulation. Abbreviations are following as TF (Transcription factor), H3K (Histon 3 Lysine), HMT (Histone methyltrasferase), HDM (Histone demethylase), and SAM (S-Adenosyl methionine).

In human cancer, abnormal DNA methylation occurs alongside a number of other types of epigenetic aberrations. Of these alterations, post-translational histone modifications are critically important in cancer cells. Chromatin is composed of DNA, histones, and various other proteins. The amino-terminal tails of some histones project out of the nucleosome core and are subject to a number of posttranslational modifications, or marks, including acetylation, phosphorylation, ubiquitination, and methylation [3]. Acetylation and methylation of histones have been intensely studied and both types of modifications can remodel chromatin and lead to controlling the functional state of chromatin. Histone acetylation and deacetylation are essential for gene regulation. Acetylation generally leads to active transcription, whereas hypoacetylation is an indicator of inactive transcription. Histone methylation can indicate both active and inactive transcription, and the state of mono-, di-, and trimethylation has different effects. Methylation is facilitated by the enzymes known as histone methyltransferases (HMTs). In the last decade, aberrant patterns of histone modifications were found to be a hallmark of cancer. Therefore, there has been a large number of studies in this field and an increasing number of histone marks have been identified. Histone modification to H3 has been very well studied and characterized so far. Some of these marks are implicated in the activation of transcription. Examples include acetylation of H3K4 (histone (H) 3 lysine (K) 4) and methylation of H3K4, H3K36, and H3K79 [4,5,6]. In contrast, other marks result in an inactive chromatin state and transcriptional repression. The primary examples of these types of modifications include methylation of H3K9, H3K27, and H4K20 (Figure 1) [7,8,9]. 

It should be noted that the H3K9 is found primarily in a gene-poor region, such as telomeres and centromeres, and is associated with X chromosome inactivation and gene repression at promoter regions [10]. On the other hand, H3K27 is generally found in gene-rich regions and acts as a temporary marker correlating with the development of regulators [11]. Hypermethylation of CpG islands in the promoter regions of tumor-suppressor genes in cancer cells is associated with a particular combination of histone markers: deacetylation of histones H3 and H4, loss of H3K4 trimethylation, and gain of H3K9 methylation and H3K27 trimethylation [1]. 

Histone methylation is carried out by any number of histone lysine methyltransferases such as those in the polycomb complex and demethylation is carried out by a number of demethylases such as LSD1 and the Jumonji-C-domain-containing proteins [12,13,14]. Global changes of these and other histone marks have been found to be present in a wide range of malignancies, suggesting that abnormalities in the chromatic state may be present in cancer cells. Genome-wide studies of histone modification have been provided to better characterize the chromatin of malignant cells by establishing the overall profile of histone modifications in cancer cells. 

### 2.2. DNA Methylation in Cancer 

Research has mostly focused on the genetic basis of cancer in terms of how mutational activation of oncogenes or inactivation of tumor suppressor genes (TSGs) support cellular control pathway changes. Since the 1990s, a growing research effort has focused on the importance of epigenetic alterations, which may also be critical heritable changes for all human cancers [1]. The primary effect of epigenetic changes in cancer depends on the stages of cancer progression, and the secondary effect of these changes is how they affect the biology of each developmental step towards invasive disease. There are three main epigenetic mechanisms as follows: DNA methylation, histone modification, and microRNAs (Figure 1) [1,15]. 

**Figure 2:**
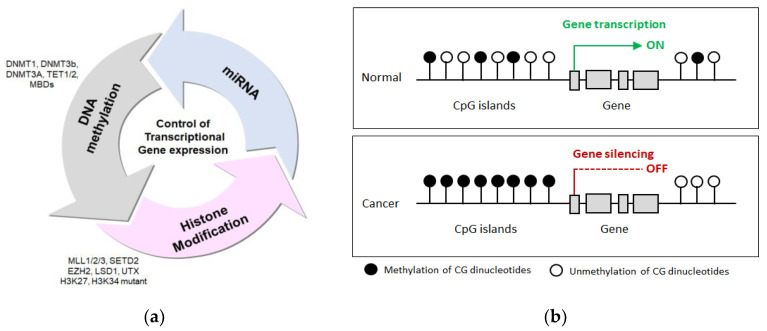
Epigenetic mechanisms in cancers. (**a**) Transcriptional gene expression, particularly gene silencing, is mainly regulated by DNA methylation, histone modifications, and microRNAs. Representative enzymes that contribute to these modifications include DNA methyltransferases (DNMTs), the TET family, methyl-CpG-binding domains (MBDs), histone modifying enzymes (MLL1/2/3, SETD2, EZH2, LSD1, and UTX), histone methyltransferases (HMTs). The relationship among these processes establishes a heritable repressive state at the start site of genes resulting in transcriptional gene silencing (Adapted from You and Jones 2012). (**b**) Promoter DNA methylation patterns in normal and tumor cells. In normal cells, CpG dinucleotides are randomly methylated and not associated with CpG islands located in the promoter region. Thus, the unmethylated status of CpG islands in gene promoters permits active gene expression. In cancer cells, CpG islands in the gene promoter region become abnormally hypermethylated, causing transcriptional silencing of genes. Circles indicate CpG dinucleotides.

In genomic DNA, methylation occurs at cytosine bases, which comprise 50% of the positions in CpG dinucleotides. CpG dinucleotides are depleted from the eukaryotic genome [16]. This review focuses mostly on studies on genes regulated by DNA methylation in colon cancer and IBD. DNA methylation is a progressive enzymatic process (1) starting with the addition of a methyl group to the 5′ carbon position of the pyrimidine ring of cytosines (C) to produce 5-methylcytosine (5mC). (2) This covalent modification is catalyzed by DNA methyltransferases (DNMTs) in CpG islands, which are mostly located in the upstream region, called the promoter region. Regardless of the transcriptional level of the gene, normal cells globally lack methylation levels at CpG islands. Therefore, if this process is interrupted, the promoters may become abnormally hypermethylated, leading to transcriptional repression [17]. In addition, methylation can induce a compact chromatin structure by supporting additional binding sites for methyl-binding proteins, which also cause gene transcriptional repression by interactions with histone deacetylases [18] (Figure 2a). 

Human cancer is the best model system for the investigation of methylation because promoter DNA hypermethylation occurs in the promoter regions of genes. This phenomenon has been established as a specific event in cancer cells that normally involves unmethylated gene promoter regions associated with transcriptional silencing by promoter hypermethylation, leading to loss of tumor suppressor gene function [19] (Figure 2b). Transcriptional silencing of tumor suppressors by promoter hypermethylation may be a critical event contributing to oncogenic development [1,2]. Most importantly, inactivation of tumor suppressors by hypermethylation can affect numerous cellular pathways, such as programmed cell death, the DNA repair system, control of the cell cycle, angiogenesis, and tumor invasion. Retinoblastoma (*Rb*), *p16*, *hMLH1,* and *VHL* are well-known tumor suppressors in cancer that are specifically silenced by CpG island hypermethylation of the promoter [19,20]. 

Colon cancer is a genetic and epigenetic disease. Evidence from the last decade has demonstrated that epigenetic alterations have a pathological role in colorectal cancer (CRC) [21]. Epigenetic alterations play a major role in the initiation and progression of CRCs, and epigenetic instability appears to be a common phenomenon in CRC. In CRC, inactivation of tumor suppressor genes by promoter hypermethylation has been observed at each pathological process [17]. Numerous genes have been reported to be hypermethylated and silenced in CRC, and some commonly well-known methylated genes include *MLH1, CDH1, TIMP3, O^6^-MGMT, SFRP1*, *SFRP2, p16, APC, HIC1, and CHFR* [19,22,23]. 

### 2.3. miRNAs in Cancer 

Sequences of microRNAs (miRNAs) are highly similar among species, and play critical roles in numerous biological processes including cell proliferation, development, differentiation, and apoptosis. In addition, subsets of miRNAs are thought to play roles as tumor suppressor genes or oncogenes, and their dysregulation is a common feature of human cancer (Figure 1) [24,25]. In human cancer, it has been known that miRNA expression profiles differ between normal tissues and cancer, as well as between different tumor types [26,27]. Importantly, the downregulation of subsets of miRNAs has been found in many of these studies, suggesting that some of these miRNAs may act as tumor suppressor genes [27]. Recent advance suggests that the mechanism underlying the downregulation of miRNA expression in cancer is associated with epigenetic alterations. Specifically, tumor suppressors of miRNAs have been investigated in more detail. For example, the first report of altered miRNA deletion and downregulated expression of miR-15 and miR-16, two miRNAs thought to target the antiapoptotic factor B cell lymphoma 2 (BCL2) in chronic lymphocytic leukemia (CLL) [28]. The downregulations of let-7 and miR-15/miR-16 and miR-127 are known to target the oncogenic factors RAS and BCL-2, respectively [29,30]. 

## 3. Promoter DNA Hypermethylation as a Biomarker for Clinical Use

**Figure 3:**
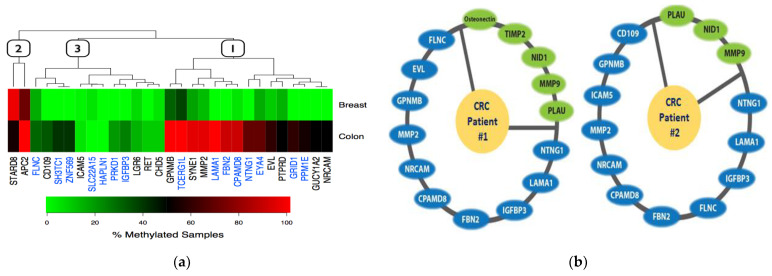
Identification of DNA hypermethylated genes in breast and colon cancers. (**a**) Heatmap cluster analysis of the 29 DNA hypermethylated genes shows three distinct gene groups (numbered blocks) identified by methylation frequency. A subset of genes was noted in our previous studies [32,35], and genes with blue letters were identified later [36]. (**b**) Multiple hypermethylated ECM genes in two primary colorectal cancers (CRC patients #1 and #2) (unpublished data).

Aberrant DNA methylation in CRC is currently receiving greater attention than histone modification due to its clinical utility as a biomarker. Recent efforts in genome-wide sequencing of CRC have identified a large number of genetic or epigenetic changes that are integrated with a few cellular signaling pathways, such as invasion, metastasis, apoptosis, and cell senescence [19,31]. Therefore, these efforts have led to new directions to discover unknown genes that are regulated by genetic or epigenetic mechanisms in CRC (Figure 3a) [32]. 

Generally, epigenome profile technologies, including the Infinium Human Methylation 850K BeadChip (Illumina Inc, San Diego, CA, USA), are high-throughput platforms that allow the methylation state of 850,000 CpGs to be assayed and analyzed [33,34]. This technology may lead to the identification of numerous newly hypermethylated genes at the genome-wide level using bioinformatics analysis. Using this recent technology to analyze the global DNA methylation level of human cancer, it is necessary to first establish a methylation profile to identify the differential pattern between two groups, such as equivalent normal and tumor tissues from the same clinical patient samples.

Identification of global genome-wide methylation profiling in CRC has broad capacity for important clinical applications, particularly molecular markers, which are becoming increasingly attractive due to pharmacological reversibility [37], thereby improving the development of insufficient current diagnostic methods. Based on the accumulation of a large amount of data from genome-wide DNA methylation profiling, many studies have recently reported DNA methylation as a biomarker for the detection or prognosis of CRC, and there are integrative and comprehensive studies on the biological significance for tumor suppressor genes as methylation biomarkers for clinical use. Numerous methylation-based biomarker candidates were identified by genome-wide transcriptome profiles and it has been found that these candidates are strongly linked by biological pathway. In fact, we have identified that multiple genes, which are components of ECM pathway, are hypermethylated in actual CRC patient samples, suggesting that the ECM pathway eventually may be inactivated by DNA hypermethylation (Figure 3b). Hypermethylated genes that have been well characterized to be associated with clinical significance in CRC are described in Table 1. The list of promising DNA methylation biomarkers in colon cancer are summarized in terms of previously reported literature, including validation data using patient samples and clinical information. Although there are many reports that implicate biomarkers for clinical use using genome-wide profiling analysis, multiple, prospective, large-scale population studies to validate biomarker candidates are necessary to prove their clinical significance as promising biomarkers. 

Analysis of DNA methylation has led to a new generation of cancer biomarkers [38]. Although certain genomic mutations provide sensitive and specific biomarkers [39,40], their utility is undermined due to their heterogeneity. DNA hypermethylation in cancers provides major advantages when designing biomarker assays due to affecting identical residues in the regulatory regions of specific genes. Accordingly, numerous studies have employed DNA methylation of specific genes for biomarker and diagnostic development [1,38,41]. Such diagnostic tests can broadly be used for early detection of cancers, assessing prognosis, determining the effects of therapy or detecting human diseases.

## 4. DNA Methylation in IBD

In the past decade, there have been integral improvements in our understanding of genetic factors that contribute to inflammatory bowel diseases (IBDs), including ulcerative colitis (UC) and Crohn’s disease (CD). 

Over decades, genetic studies have provided many susceptibility candidate loci, and innate and acquired immune responses have been implicated in IBD pathogenesis [42]. Thus, recent international collaboration studies have provided critical evidence that genetic changes affect IBD pathogenesis, causing abnormal immune responses. However, identified genetic factors account for only a limited portion of the disease variance (13.6% for CD and 7.5% for UC), which covers only approximately 20% of the genetic risk [43,44,45]. However, genetic factors may explain a part of IBD pathogenesis, indicating a need to better understand the interaction of genes and the environment during IBD development. Epigenetic factors may explain these interactions between the environment and the genome. Epigenetic studies may provide a new approach to understanding the pathogenesis of IBD, suggesting that IBD is a genetic and epigenetic disease. 

The first step of DNA methylation studies has mostly focused on the relationship between cancer and IBD. Several reports have suggested that promoter hypermethylation of multiple genes is associated with UC [46]. Other studies have identified many kinds of genes, such as *CDH1*, *p16*, *MDR1*, and *GDNF*, which are hypermethylated with high frequencies in patients with UC. Promoter hypermethylation of the *CDH1* gene has been confirmed to be associated with long-standing inflammation. Thus, the DNA methylation of this gene, as a useful biomarker, may be implicated in patients with UC for detecting patients at high risk for developing colorectal cancer [47]. Recently, we confirmed that several genes known to be hypermethylated in the early stage of CRC are hypermethylated in Korean UC patients [48]. However, further study is necessary to define the clinical relevance, such as disease duration, severity, extent, phenotype, and activity state of inflammation and dysplasia. 

Growing evidence suggests that there are significant differential DNA methylation patterns between normal and IBD patient samples [49,50]. To understand the molecular basis of CD, comprehensive genome-wide studies identifying a number of diverse susceptibility loci associated with CD pathogenesis have been performed [45]. Although little is known about DNA methylation patterns in CD pathogenesis, we recently reported that the *TCERG1L* gene is hypermethylated in serum samples from CD patients, suggesting that DNA methylation is an important mechanism to understand CD pathogenesis [50]. 

**Table 1:** Possible promising DNA methylation biomarkers in colorectal diseases.

Diseases Types	Gene	Samples for Study	Methylation Sensitivity	References
CRC	*TFPI2*	Tumor tissue	99%	Grockner et al., 2009 [51]
		Stool	73%	Grockner et al., 2009 [51]
	*FBN2*	Tumor tissue	86%	Yi et al., 2012 [52]
	*TCERG1L*	Tumor tissue	99%	Yi et al., 2012 [52]
	*SEPT9*	Plasma	69%	Lofton-Day et al., 2008 [53]
	*p16*	Serum	70%	Nakayama et al., 2008 [54]
	*EVL*	Tumor tissue	60%	Yi et al., 2011 [36]
	*IGFBP3*	Tumor tissue	25%	Yi et al., 2011 [36]
	*NDRG4*	Tumor tissue	86%	Melotte et al., 2009 [55]
		Stool	61%	Melotte et al., 2009 [55]
UC	*FAM217B,*	Tissues	62%	Kang et al., 2016 [56]
	*KIAA1614*	Tissues	64%	Kang et al., 2016 [56]
	*RIB2*	Tissues	91%	Kang et al., 2016 [56]
	*SYNE1*	Tissues	80%	Papadia et al., 2014 [57]
	*FOXE1*	Serum	60%	Papadia et al., 2014 [57]
CD	*TCERG1L*	Serum	57%	Bae et al., 2014 [50]
	*FHIT*	Tissues	71%	Kim et al., 2020 [58]
IBD	*TGFB2*	Tissues	30%	Azuara et al., 2013 [59]
	*SLIT2*	Tissues	65%	Azuara et al., 2013 [59]
	*TMEFF2*	Tissues	25%	Azuara et al., 2013 [59]
	*ITGA4*	Tissues	80%	Gerecke et al., 2015 [60]
	*TFPI2*	Tissues	30%	Gerecke et al., 2015 [60]

## 5. Early Detection Methylation Biomarkers in CRC

Because early detection of human diseases has led to an improved clinical outcome for multiple types of cancer, numerous studies have focused on the development of early detection strategies. DNA methylation changes occur in the early stage of cancer development and are potentially great early indicators of existing human disease as well as risk assessment for the development of disease [61]. 

### 5.1. TFPI2

The tissue factor pathway inhibitor (*TFPI2*) gene is at the intersection of both the hypermethylome and PcG-marked genes. There are several studies on the biological roles of *TFPI2*, a Kunitz-type serine proteinase inhibitor, associated with protecting the extracellular matrix of cancer cells from degradation [62]. In addition, it has been suggested that loss of TFPI2 function may predispose cells towards a proinvasive program, such as in late stages of carcinogenesis. Our previous study on the CRC “DNA hypermethylome” identified *TFPI2* hypermethylation based on an expression array-based approach [32]. Aberrant promoter hypermethylation of *TFPI2* was detected in almost all CRC adenomas (97%, n = 56) and stages I to IV CRCs (99%, n = 115). Therefore, in CRC data using both tumor tissues from patients and stool DNA, *TFPI2* has been strongly suggested as a potential biomarker for noninvasive detection of colorectal neoplasia [51]. Since *TFPI2* has been identified as an early detection marker of CRC, growing evidence has suggested this gene as a potential biomarker in other types of cancer, such as gastric [63] lung [64], pancreas [65], oral [66], esophageal [67], and liver cancer [68].

### 5.2. FBN2 and TCERG1L

Our previous study identified hypermethylation of the *FBN2* and *TCERG1L* genes [32]. Both genes are frequently hypermethylated (>60%) in adenomas (tubular adenoma and villous adenoma). Fibrillin 2 (FBN2) is an extracellular matrix protein, and the transcription elongation regulator 1-like (*TCERG1L*) gene is located on chromosome 10 and has recently been shown to have frequent cancer-specific methylation according to our microarray-based approaches [32]. Although little is known about their biological function regarding epigenetic changes in human cancers, the methylation of these genes has great potential to detect early-stage colon cancer.

## 6. Prognostic Methylation Biomarkers in CRC

### 6.1. EVL and IGFBP3

**Figure 4:**
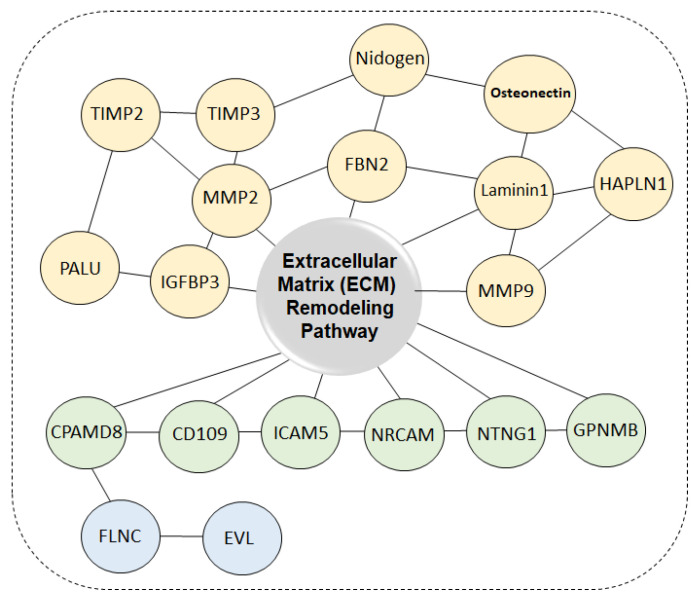
Schematic representation of extracellular matrix (ECM) pathway silencing by DNA hypermethylation in colon cancer. Six genes, namely, *TIMP2*, *PLAU, TIMP3, Osteonectin*, *MMP9*, and *Nidogen*, are regulated by promoter DNA hypermethylation in other cancer types and have been shown to be similarly altered in CRC in our previous study [36]. In addition, Yi et al. identified genes within the extracellular matrix, including 13 hypermethylated genes in CRC derived from our gene discovery approach (*IGFBP3, HAPLN1*, *ICAM5*, *CD109*, *FLNC*, *GPNMB*, *NRCAM*, *EVL*, *NTNG1*, *MMP2*, *LAMA1*, *CPAMD8*, and *FBN2*). Different colors indicate the locations of each gene. Yellow circles, green color and blue color indicate ECM, membrane, and cytoplasm, respectively. The functional gene ontology analysis was based on the MetaCore database (Adapted from Yi et al. 2011).

By merging genome-wide genomic and epigenomic change profiles, new genes have been identified, and core pathways associated with these genes have been defined in CRC. We previously emphasized that DNA hypermethylation can affect many new genes associated with key pathways altered in CRC. An integrative and comprehensive approach of multiple whole genome analyses (genetic and epigenetic) has been used to define the core pathway, namely, the extracellular matrix (ECM) pathway, which is silenced in all colon cancers. Simultaneous DNA hypermethylation of a subset of genes that are major components of the ECM remodeling pathway is also significantly associated with poor survival in adjusted analyses of CRC patients. In addition, the promoter hypermethylation of both *EVL* and *IGFBP3* has been identified as novel methylation biomarkers, suggesting that these both gene methylation is associated with worse survival of CRC patients. Taken together, the methylation of *IGFBP3* and *EVL* might be potentially useful in defining prognostic biomarkers for CRC patients (Figure 4) [36].

### 6.2. SEPT9

Multiple studies have studied the use of single or combined DNA methylation-based biomarkers for diagnostic purposes for CRC. Several groups have identified methylation of the *SEPT9* gene as one of the best candidates as a prognostic biomarker in different CRC cohorts [69,70]. Importantly, de Vos et al. developed and expanded a method to validate the *SEPT9* blood-based biomarker assays in plasma samples of CRC patients [71], which support the strong evidence about the use of biomarkers for the detection of CRC using less invasive screening methods. 

### 6.3. Vimentin

Although numerous studies have reported the use of single or combined DNA methylation-based biomarkers in cancer, it is suggested that testing combined genes is more useful for diagnostic or prognostic rather than using a single gene. Ahmed et al. identified a highly methylated gene panel in CRC including *VIM* to implicate their clinical use for prognostic methylation biomarkers [69]. Subsequently, the promoter methylation of Vimentin *(VIM)* has been validated as promising biomarkers to detect CRC in patients [69,72]. Recently, there has been an effort to identify an effective methylation biomarker; a methylation methyl-beaming assay has been developed to detect the methylation of *VIM* in plasma samples of CRC patients [73]. Using *VIM* gene methylation, this technology achieved 59% sensitivity in early stage of CRC. *SEPT9* and *VIM* are the only methylation markers being used because of the multicenter retrospective and prospective validations performed on these biomarkers.

### 6.4. NDRG4

Melotte et al. identified N-Myc downstream-regulated gene 4 (*NDRG4*) as a potential methylation biomarker in CRC [55]. *NDRG4* was originally identified by screening the gene expression change profile in the tumor endothelium by a microarray approach [74]. *NDRG4* is frequently hypermethylated in CRC patient samples (>70%) along with adenomas compared to noncancerous colon mucosa (4%). To understand the clinical relevance of utility as a methylation biomarker, the promoter methylation of *NDRG4* has been tested in stool DNA from CRC patient samples. These experiments provide strong evidence that the promoter methylation of *NDRG4* may have potential utility as a noninvasive biomarker to screen the risk of CRC.

## 7. Methylation Biomarkers in IBD

### 7.1. FOXE1 and SYNE1

Patients with UC, which is a chronic inflammation, have a higher risk of developing CRC [75]. The disease duration of UC is an increasing risk factor for the development of CRC. *SYNE1* and *FOXE1* are two genes that have been recently linked to tumor growth, especially in gastrointestinal cancer. Hypermethylation of these genes has been studied in CRC patients, and these genes are being explored as noninvasive biomarkers for the detection of colorectal cancer in stool and blood samples [76]. Interestingly, Papadia et al. found that *SYNE1* and *FOXE1* hypermethylation events frequently occur in colitis-associated colorectal cancer, suggesting a useful marker of neoplasia in long-standing IBD [57].

### 7.2. TCERG1L

As mentioned above, the *TCERG1L* gene is hypermethylated in the early stage of CRC. Bae et al. hypothesized that methylation of the *TCERG1L* gene can be detected in serum samples from patients with CD [50]. In a cohort of CD patients, hypermethylation of *TCERG1L* has been detected at high frequencies (57%) in sera, suggesting a potential noninvasive biomarker to reduce the risk or prevent the progression of advanced stages of disease [50]. However, due to the lack of testing *TCERG1L* methylation in control samples and clinical data of patients with CD, including disease duration, further studies are necessary to define the clinical relevance of *TCERG1L* in patients with CD.

### 7.3. FAM217B, KIAA1614, and RIB2

To identify new genes regulated by promoter hypermethylation in IBD, genome-wide DNA methylation profiling was performed using UC patient samples compared to normal colon tissues. Differential methylation patterns were identified between normal colon tissues and patient samples with UC. Regarding promoter hypermethylation in terms of correlation with transcriptional gene expression, 48 genes were identified in this approach. However, these genes should be validated in cohort samples with UC to prove epigenetic regulation in patients with CD. After strong validation by RT-PCR and MSP in clinical samples, *FAM217B, KIAA1614,* and *RIB2* were found to be hypermethylated in patients with UC in a disease-specific manner, indicating lower methylation of these genes in normal colon tissues. Kang et al. suggested that this novel hypermethylated gene panel could be a useful molecular biomarker for surveillance of UC patients, implicating their diagnosis or prognosis [56]. 

### 7.4. FHIT

Using the same approach by genome-wide DNA methylation profiling in patients with CD, Kim et al. identified many new hypermethylated genes in patients with CD. However, after validation using methylation analyses, the fragile histidine triad (*FHIT*) gene was identified to be frequently hypermethylated in patients with CD. Aberrant transcripts of *FHIT* have been reported in multiple cancer types, such as colon, gastric, esophageal, and lung cancers [77,78,79,80]. Detection of promoter methylation of *FHIT* has been identified by meta-analyses and demonstrated to be useful for the early diagnosis of breast and NSCLC carcinomas [81,82]. Although the biological function of *FHIT* has been implicated to be associated with tumor progression, to the best of our knowledge, this was the first report implicating aberrant DNA hypermethylation of *FHIT* in IBD pathogenesis.

## 8. Conclusions and Future Directions

World-wide consortia, such as the International Cancer genome consortium or the Cancer Genome Atlas (TCGA), provide comprehensive genomic or epigenomic data of various cancer types for many laboratories around the world. In recent years, much effort has led to a better understanding of the mechanisms that underlie DNA methylation changes in human cancer and other diseases. The value of epigenetic changes as candidate biomarkers is reflected in thousands of scientific studies published to date that associate DNA methylation with clinical relevance. 

In the last decade, DNA methylation markers have been established as the most promising clinical utilities due to their power of diagnostics, and they will provide a tool for risk assessment, early detection, molecular diagnostics of resected specimens, predicting chemotherapy, and monitoring disease recurrence. Considering the development of methylation biomarkers in cancers and other diseases, it is important to understand not only that the identification of new genes is regulated by epigenetic mechanisms, but also that the development and application of sophisticated technology, such as imaging, nanoparticle-enabled, noninvasive, and minimally invasive technology, are important to confirm the diagnosis of human diseases. This review focused on the discovery of epigenetic alterations in colorectal disease, which may lead to the exploration of their future clinical applications as molecular biomarkers or potential therapeutic targets in colorectal diseases. 

At last, the combination and integration of epigenomics, genomics, and all the other ‘omics’ such as transcriptomic, proteomic, and metabolomics aspects, will be essential to maintain the increasingly rapid progress towards a full understanding of the underlying molecular mechanisms that regulate the initiation and development of cancer progress. Furthermore, these cancer genomic or epigenomic signatures will help us identify new potential prognostic and detection tools and eventually, to develop effective clinical therapies.

## Data Availability

Not applicable.
